# Identification and Verification of Potential Biomarkers in Gastric Cancer By Integrated Bioinformatic Analysis

**DOI:** 10.3389/fgene.2022.911740

**Published:** 2022-07-15

**Authors:** Chenyu Sun, Yue Chen, Na Hyun Kim, Scott Lowe, Shaodi Ma, Zhen Zhou, Rachel Bentley, Yi-Sheng Chen, Margarita Whitaker Tuason, Wenchao Gu, Chandur Bhan, John Pocholo Whitaker Tuason, Pratikshya Thapa, Ce Cheng, Qin Zhou, Yanzhe Zhu

**Affiliations:** ^1^ AMITA Health Saint Joseph Hospital Chicago, Chicago, IL, United States; ^2^ Department of Clinical Medicine, School of the First Clinical Medicine, Anhui Medical University, Hefei, China; ^3^ College of Osteopathic Medicine, Kansas City University, Kansas City, MO, United States; ^4^ Department of Epidemiology and Health Statistics, School of Public Health, Anhui Medical University, Hefei, China; ^5^ Menzies Institute for Medical Research, University of Tasmania, Hobart, TAS, Australia; ^6^ Department of Orthopedics, Shanghai General Hospital, Shanghai Jiao Tong University School of Medicine, Shanghai Jiao Tong University, Shanghai, China; ^7^ Faculty of Medicine and Surgery, University of Santo Thomas, Metro Manila, Philippines; ^8^ Department of Diagnostic Radiology and Nuclear Medicine, Gunma University Graduate School of Medicine, Maebashi, Japan; ^9^ The University of Arizona College of Medicine, Tucson, AZ, United States; ^10^ Banner-University Medical Center South, Tucson, AZ, United States; ^11^ Mayo Clinic, Rochester, MN, United States; ^12^ Department of Oncology, The First Affiliated Hospital of Anhui Medical University, Hefei, China

**Keywords:** gastric cancer, hub genes, bioinformatics analysis, meta-analysis, ROC (receiver operating curve), prognostic value, immune infiltration

## Abstract

**Background:** Gastric cancer (GC) is a common cancer with high mortality. This study aimed to identify its differentially expressed genes (DEGs) using bioinformatics methods.

**Methods:** DEGs were screened from four GEO (Gene Expression Omnibus) gene expression profiles. Gene ontology (GO) and Kyoto Encyclopedia of Genes and Genomes (KEGG) analyses were performed. A protein–protein interaction (PPI) network was constructed. Expression and prognosis were assessed. Meta-analysis was conducted to further validate prognosis. The receiver operating characteristic curve (ROC) was analyzed to identify diagnostic markers, and a nomogram was developed. Exploration of drugs and immune cell infiltration analysis were conducted.

**Results:** Nine up-regulated and three down-regulated hub genes were identified, with close relations to gastric functions, extracellular activities, and structures. Overexpressed Collagen Type VIII Alpha 1 Chain (COL8A1), Collagen Type X Alpha 1 Chain (COL10A1), Collagen Triple Helix Repeat Containing 1 (CTHRC1), and Fibroblast Activation Protein (FAP) correlated with poor prognosis. The area under the curve (AUC) of ADAM Metallopeptidase With Thrombospondin Type 1 Motif 2 (ADAMTS2), COL10A1, Collagen Type XI Alpha 1 Chain (COL11A1), and CTHRC1 was >0.9. A nomogram model based on CTHRC1 was developed. Infiltration of macrophages, neutrophils, and dendritic cells positively correlated with COL8A1, COL10A1, CTHRC1, and FAP. Meta-analysis confirmed poor prognosis of overexpressed CTHRC1.

**Conclusion:** ADAMTS2, COL10A1, COL11A1, and CTHRC1 have diagnostic values in GC. COL8A1, COL10A1, CTHRC1, and FAP correlated with worse prognosis, showing prognostic and therapeutic values. The immune cell infiltration needs further investigations.

## Background

As one of the top five common malignancies, gastric cancer (GC) is the fourth leading cause of cancer-associated death around the globe, according to Global cancer statistics 2020. It accounted for more than one million new cases in 2020 and is responsible for one in every 13 deaths globally (Sung et al., 2021). The incidence and mortality of GC vary among different populations and geographical locations. GC is the leading cause of cancer death in several South Central Asian countries. Incidence rates are highest in Eastern Asia and Eastern Europe, while the incidence rates are generally low in Northern America, Northern Europe, and African regions (Sung et al., 2021). These differences could be attributed to the various environmental risk factors, such as the different prevalence of Helicobacter pylori (H. pylori) infections, alcohol consumptions, tobacco smoking, consumption of preserved salty food and processed meat, ingestion of grilled or barbecued meat and fish, and viral infection (Hooi et al., 2017; Research. WCRFAIfC, 2018; Palrasu et al., 2021; Sung et al., 2021). In addition to these environmental factors, genetic factors were also thought to affect the carcinogenesis of GC, as less than 5% of H. pylori infected hosts will develop GC, and evidence of genetic alterations, such as aberrantly expressed activation-induced cytidine deaminase (AID), has emerged (Nagata et al., 2014; Nakanishi et al., 2021). Significant progress in the diagnosis and treatment of GC has been made, such as development of novel human epidermal growth factor receptor 2 (HER2)-targeted drugs for GC (Zhu et al., 2021), the development of minimally invasive surgery, and endoscopic mucosal resection (EMR) and endoscopic submucosal dissection (ESD) (Roh et al., 2020; Wang et al., 2021). Moreover, several biomarkers have been found for the target therapy of GC, such as programmed death 1 (PD-1), HER2, and MNNG HOS transforming gene (MET) (Choi et al., 2022). PD-1 is an inhibitory checkpoint receptor protein expressed on cytotoxic T cells and other immune cells (Pardoll, 2012). Some tumor cells express high levels of PD-L1 to evade from immune system, as PD-1/PD-L1 interaction induces cytotoxic T cell inactivation and downregulation of immune responses (Schreiber et al., 2011), and PD-L1 expression was proposed to be a potential biomarker of response to pembrolizumab (Ghidini et al., 2021). HER2 overexpression is particularly important in GC, as targeted therapy trastuzumab has been widely used to treat HER2+ GC (Bang et al., 2010). MET activation triggers a downstream cascade of phosphoinositide 3-kinases (PI3K) and Rat sarcoma virus (RAS) signaling and regulates cell survival and proliferation (Zhang et al., 2018), Thus, the over-activation of MET plays a critical role in cancer development and is frequently identified in various types of tumors, including GC (Ariyawutyakorn et al., 2016). However, the prognosis of GC remains quite unsatisfactory due to its low early diagnosis rate, with a 5-year overall survival (OS) of less than 40% (Tan, 2019). Therefore, exploration of novel biomarkers that are sensitive and specific for early diagnosis, as well as predictors of prognosis and response to potential targeted treatment, is pivotal in the management of GC.

With the development of next-generation sequencing (NGS) and other techniques, the availability of information related to these potential biomarkers and knowledge of the relevant gene expressions available for various tumors have increased significantly (Behjati and Tarpey, 2013; Levy and Myers, 2016; Giunchi et al., 2021; Shirdarreh et al., 2021). As a result, the mechanisms of various cancers and other diseases have become more widely studied based on bioinformatic analysis, a field combining molecular biology and information technology. Bioinformatics methods, such as data-mining, are now commonly used to explore the carcinogenesis at the molecular level, and to explore biomarkers for potential diagnostic markers, prognostic predictors, and therapeutic targets (Li et al., 2018a; Shen et al., 2019; Yang et al., 2019). As several microarray profiling studies have been performed in GC, this study integrating publicly available data of some of these existing studies, to search for the differentially expressed genes (DEGs) and ultimately biomarkers that could show potential diagnostic values, predict prognosis, and those that might become therapeutic targets.

## Methods

### Data Collection

Four gene expression profiles [GSE13911 (D'Errico et al., 2009; Wang et al., 2012a), GSE1982626, GSE54129, and GSE79973 (He et al., 2016; Jin et al., 2017)] were downloaded from the GEO database. The patient’s data from GEO datasets were obtained if the pathological sample/biopsy of the gastric cancer in the experiment group or normal gastric tissues in control group were used. GSE13911 dataset included 38 cancer tissues and 31 non-cancerous tissues, whilst GSE19826 dataset included 12 cancer tissues and 12 non-cancer tissues, GSE54129 dataset included 111 cancer tissues and 21 non-cancer tissues, and GSE79973 dataset included 10 cancer tissues and 10 non-cancer tissues. All datasets were based on GPL570 [HG-U133_Plus_2] Affymetrix Human Genome U133 Plus 2.0 Array.

### Identification of DEGs

The linear models for microarray data (LIMMA) package (Ritchie et al., 2015) based on R software was utilized to screen up-regulated DEGs within the adj. *p* < 0.01 and Log2FC > 2, and down-regulated DEGs within the adj. *p* < 0.01 and Log2FC < -2 between samples in cancer group and non-cancer group. To identify overlapping DEGs, a Venn diagram was constructed using the bioinformatics & evolutionary genomics website (http://bioinformatics.psb.ugent.be/webtools/Venn/). Volcano plot of the four datasets was drawn by Hiplot (https://hiplot.org).

### Protein–Protein Interaction Network Construction

PPI was conducted by using the Search Tool For the Retrieval of Interacting Genes (STRING) database (Szklarczyk et al., 2019), and Cytoscape software (Shannon et al., 2003). The network was constructed based on setting the medium confidence as >0.4 in the STRING database, and then imported the network into Cytoscape software for further analysis. Hub genes were selected based on plugin Cytohubba to identify hub genes through 12 algorithms (Chin et al., 2014; Zhang et al., 2019a). The top 15 genes ranked by score from each algorithm were extracted and mutual genes that were overlapped in all 12 algorithms were selected as hub genes. PPI network of the hub genes was visualized by GeneMANIA (http://www.genemania.org) (Vlasblom et al., 2015).

### Validation of Hub Genes: Survival Analysis, Expression Analysis, and Receiver Operating Characteristic Curve Analysis

Survival analysis and direct tumor/normal differential expression were conducted for the selected hub genes *via* data obtained from The Cancer Genome Atlas (TCGA) dataset (https://portal.gdc.cancer.gov/). Log2 transformed FPKM (fragments per kilobase exon-model per million reads mapped) were used. Images of immunohistochemical (IHC) staining for the protein expressed by up-regulated genes were obtained from the Human Protein Atlas (HPA) (http://www.proteinatlas.org/) to evaluate their expressions in GC (Pontén et al., 2011; Zhu et al., 2022). The Kaplan–Meier (KM) survival analysis with log-rank test was also used to compare the OS difference between the high expression and low expression group. KM curves, with *p*-values and hazard ratio (HR) with 95% confidence interval (CI), generated by log-rank tests and univariate Cox proportional hazards regression were performed using R software version v3.6.3 (The R Foundation for Statistical Computing, 2020) with “survminer,” and “survival” packages. Expression analysis was performed by using Wilcoxon rank sum test, and visualized by “ggplot2” package of R software. *p* < 0.05 was considered as statistically significant. Then, genes with significant worse overall survival (OS) were also verified by Kaplan–Meier Plotter (Szász et al., 2016).

The best discriminate cut-off point of overexpressed DEGs between the high and low expression groups were assessed by the receiver operating characteristic (ROC) curve and area under the curve (AUC) values, based on data obtained from TCGA. Log2 transformed FPKM were used. R software with “pROC” and “ggplot2” packages were used.

### Nomogram Development

A predictive model was established to predict the mortality risk based on the overexpressed hub genes with worst outcomes and other potential predictors (Iasonos et al., 2008; Balachandran et al., 2015; Liu et al., 2018a). A nomogram was developed based on the results of multivariate Cox proportional hazards analysis through “rms” and “survival” R packages. Data were obtained from TCGA. The nomogram provided a graphical representation of the factors to calculate the risk of mortality at 1, 3,and 5-year time points for an individual patient by the points associated with each risk factor. C-index was also calculated to assess the discriminatory performance of the model (Liu et al., 2018a; Kramer and Zimmerman, 2007; Pencina and D'Agostino, 2004).

### Exploration of Potential Drugs That Are Interacted With Hub Genes That Were Associated With Poor Prognosis in LC

We explored potential drugs that are interacted with hub genes linked to poor prognosis explored by using RNAactDrug (http://bio-bigdata.hrbmu.edu.cn/RNAactDrug/index.jsp), which is a comprehensive database for exploring associations between drug sensitivity and RNA molecules at expression level and other molecular levels from integrated analysis of three large-scale pharmacogenomic databases (GDSC, CellMiner and CCLE) (Dong et al., 2020).

### Go Enrichment and KEGG Pathway Analysis

The overlapping up-regulated and down-regulated DEGs were analyzed by Gene Ontology (GO) and Kyoto Encyclopedia of Genes and Genomes (KEGG) in the Database for Annotation, Visualization, and Integrated Discovery (DAVID) database (https://david.ncifcrf.gov/summary.jsp) (Huang et al., 2009a; Huang et al., 2009b). GO enrichment analysis predicted the function based on biological processes (BP), cellular components (CC), and molecular functions (MF), while KEGG analysis determined the related pathways of hub genes and their associated interactors. The results of GO and KEGG analyses were visualized by the bioinformatics online tool (http://www.bioinformatics.com.cn) (Wang et al., 2020a; Zhang et al., 2021).

### Immune Cell Infiltration of the Hub Genes With Worse Prognosis in GC

The infiltration of different immune cells and their clinical impact were assessed *via* Tumor Immune Estimation Resource (TIMER) (https://cistrome.shinyapps.io/timer/), an online tool for comprehensive investigation of molecular characterization of tumor-immune interactions based on 10,897 tumors from 32 cancer types (Li et al., 2016; Li et al., 2017). Hub genes that were associated with poor prognosis were entered into the “Gene module” to generate plots for analyzing the correlation between their expressions and immune infiltration level in GC. Positive correlation was considered for the cuff value of Cor >0.2 and *p* < 0.05 (Liu et al., 2021; Zhong et al., 2021).

### Meta-Analysis to Verify the Results of Survival Analysis of the Hub Genes

Meta-analysis was conducted to verify the results of the survival analysis of the hub genes associated with poor prognosis. Electronic databases including China National Knowledge Infrastructure database (CNKI), Web of science, and PubMed were searched to find eligible articles for conducting a meta-analysis to explore and verify survival analyses of the hub genes that were associated with worse prognosis in gastric cancer, namely, Collagen Triple Helix Repeat Containing 1 (CTHRC1). The search strategy included the following: (CTHRC1 OR collagen triple helix repeat containing 1 OR COL8A1 OR collagen type VIII alpha 1 chain OR COL10A1 OR collagen type X alpha 1 chain OR FAP OR fibroblast activation protein) AND (gastric cancer OR stomach adenocarcinoma OR gastric adenocarcinoma OR stomach cancer OR STAD). For CNKI database, the corresponding Chinese expression was used. The meta-analysis was conducted according to the Preferred Reporting Items declared by the Systematic Review and Meta-Analysis (PRISMA) (Page et al., 2021). Finally, the HR estimate with 95% CI was calculated using the effect values extracted from the incorporated articles and the corresponding result of our survival analysis. Q test and *I* ^*2*^ statistics were used to evaluate the extent of heterogeneity across the studies (DerSimonian and Laird, 1986). If significant heterogeneity (*I* ^*2*^ statistic >50% or Q test <0.1) was observed, then a random-effects model was used, otherwise a fixed-effects model was applied for combined HRs (Borenstein et al., 2010). Sensitivity analysis was performed by switching between fixed and random effects models, for testing the stability of the study results (Hernandez et al., 2020; Dan Song and Zhang, 2021). All statistical analyses were performed using STATA software (version 15.0).

## Results

### Identification of DEGs

**FIGURE 1:**
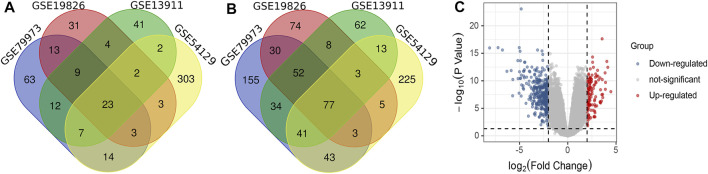
**(A)** Venn diagram of overlapped up-regulated differentially expressed genes (DEGs); **(B)** Venn diagram of overlapped down-regulated DEGs; **(C)** Volcano plot of gene expression profiles in non-cancer compared to cancer groups.

As shown in Figure 1A, there were 63 up-regulated DEGs from GSE79973, 31 DEGS from GSE19826, 41 DEGs from GSE19311, and 303 DEGs from GES54129. Among them, there were 23 up-regulated overlapping DEGs. As shown in Figure 1B, there were 155 down-regulated DEGs from GSE79973, 74 DEGS from GSE19826, 62 DEGs from GSE19311, and 225 DEGs from GES54129. Among them, 77 down-regulated overlapping DEGs were found. Volcano plot of gene expression profile in non-cancer compared to cancer groups of the four GSE datasets is shown in Figure 1C.

### PPI Network Construction and Identification of Hub Genes

**FIGURE 2:**
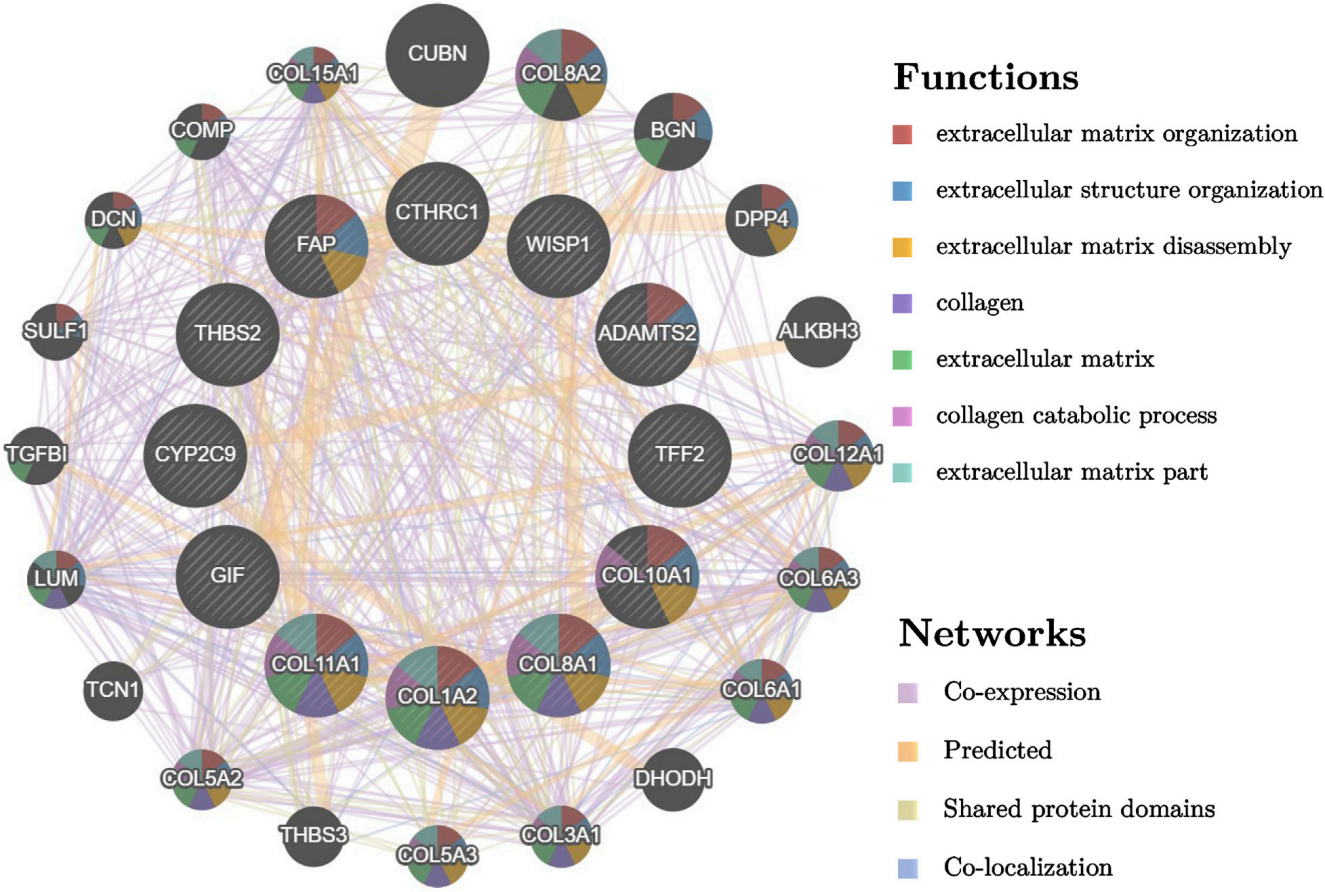
PPI network of different expressed hub genes and their interactors visualized by GeneMANIA.

Based on the STRING database and Cytoscape software, PPI networks of up-regulated overlapping DEGs (22 nodes and 46 edges) and down-regulated DEGs (71 nodes and 65 edges) were constructed. Up-regulated hub genes included Collagen Type I Alpha 2 Chain (COL1A2), thrombospondin 2 (THBS2), Collagen Type XI Alpha 1 Chain (COL11A1), Collagen Type VIII Alpha 1 Chain (COL8A1), Collagen Type X Alpha 1 Chain (COL10A1), ADAM Metallopeptidase With Thrombospondin Type 1 Motif 2 (ADAMTS2), CTHRC1, fibroblast activation protein (FAP), and WNT1-inducible-signaling pathway protein 1 (WISP1), and down-regulated hub genes included Trefoil Factor 2 (TFF2), gastric intrinsic factor (GIF), and Cytochrome P450 family 2 subfamily C member 9 (CYP2C9). PPI network of these hub genes were visualized via GeneMANIA as shown in Figure 2.

### Validation of Hub Gene Expression Analysis and Survival Analysis

**FIGURE 3:**
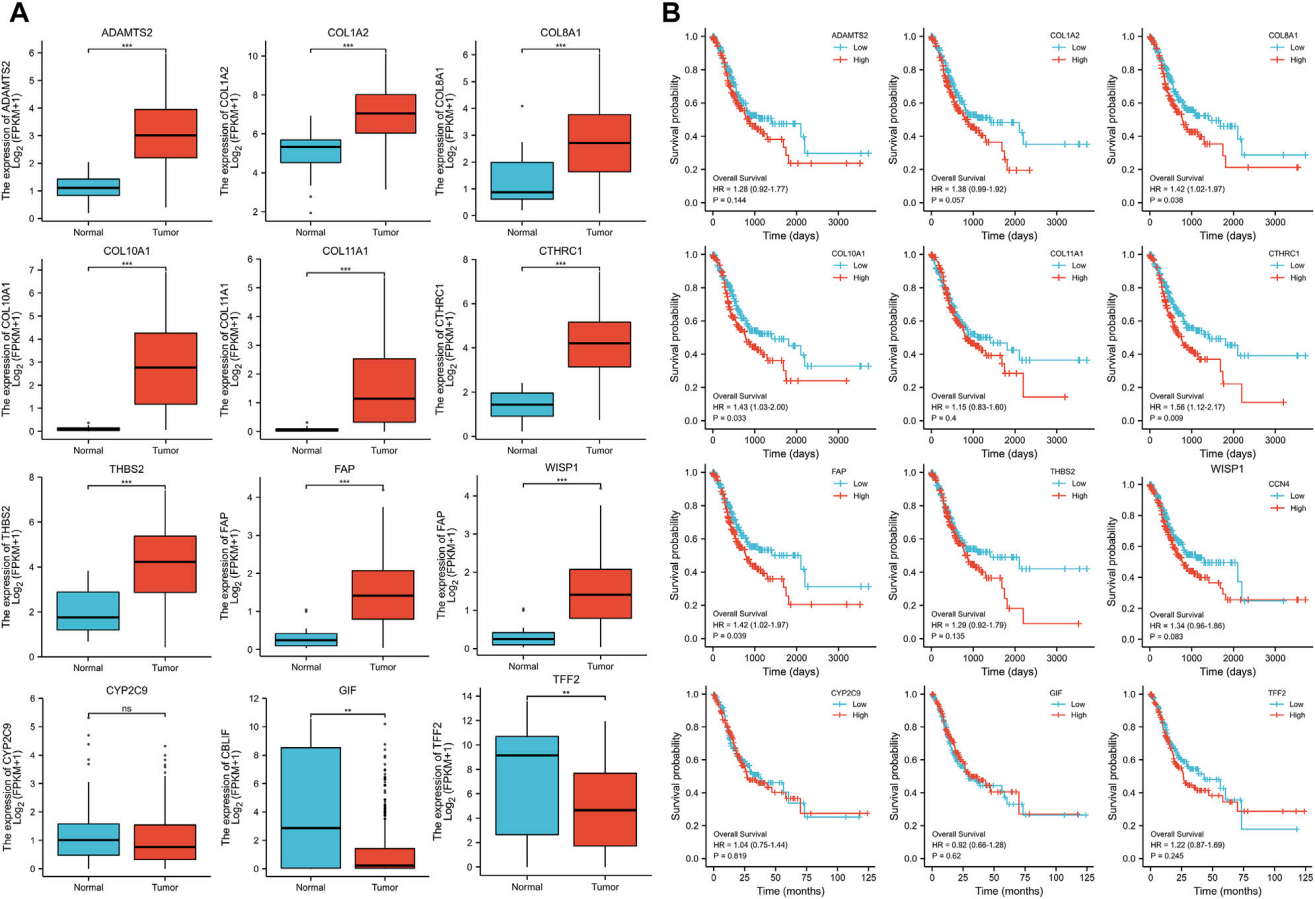
**(A)** Expression levels of the hub genes between cancer samples and normal gastric tissues **(B)** Survival analysis of the hub genes.

**FIGURE 4:**
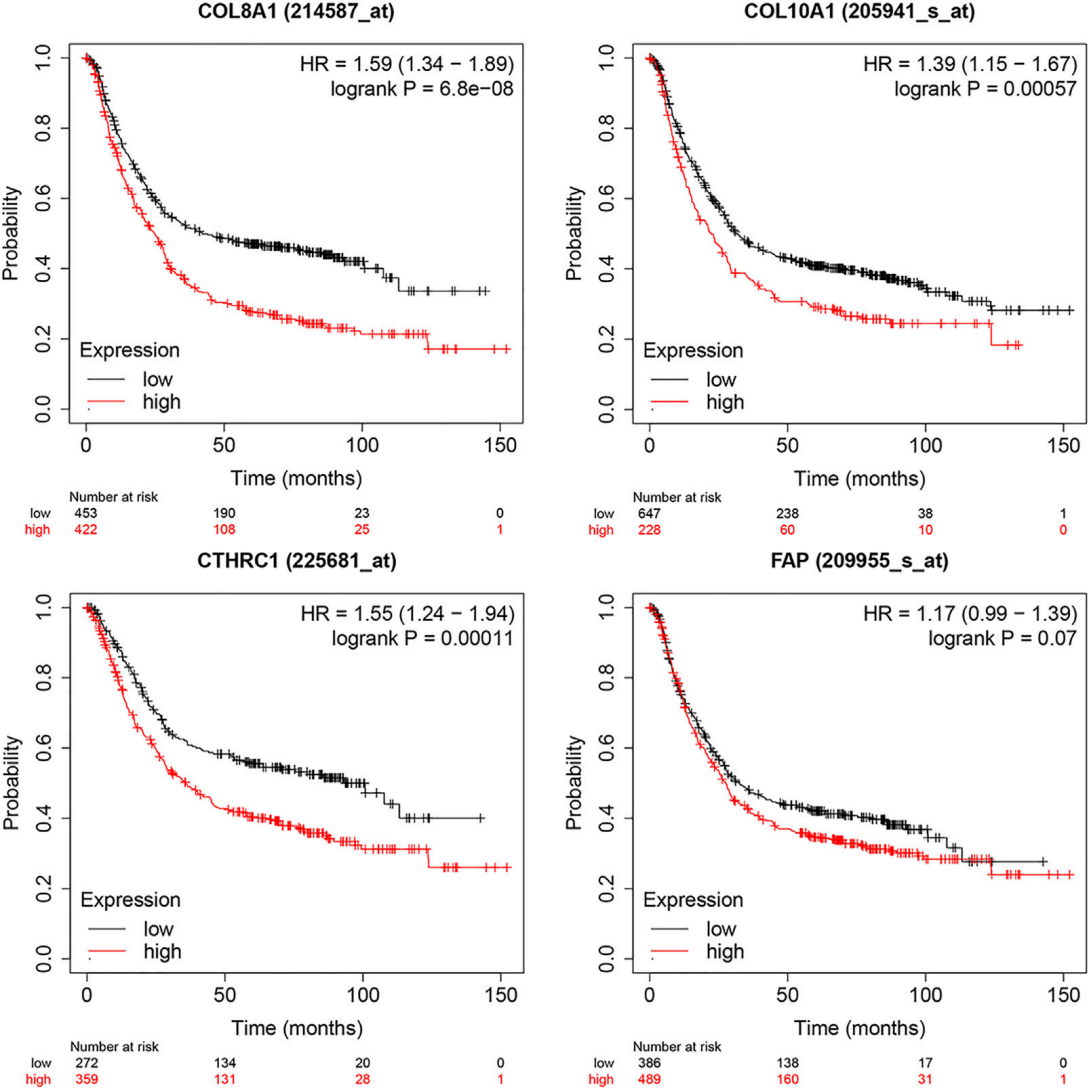
Survival analysis of Collagen Type VIII Alpha 1 Chain (COL8A1), Collagen Type X Alpha 1 Chain (COL10A1), Collagen Triple Helix Repeat Containing 1 (CTHRC1), and Fibroblast Activation Protein (FAP) by Kaplan–Meier Plotter.

**FIGURE 5:**
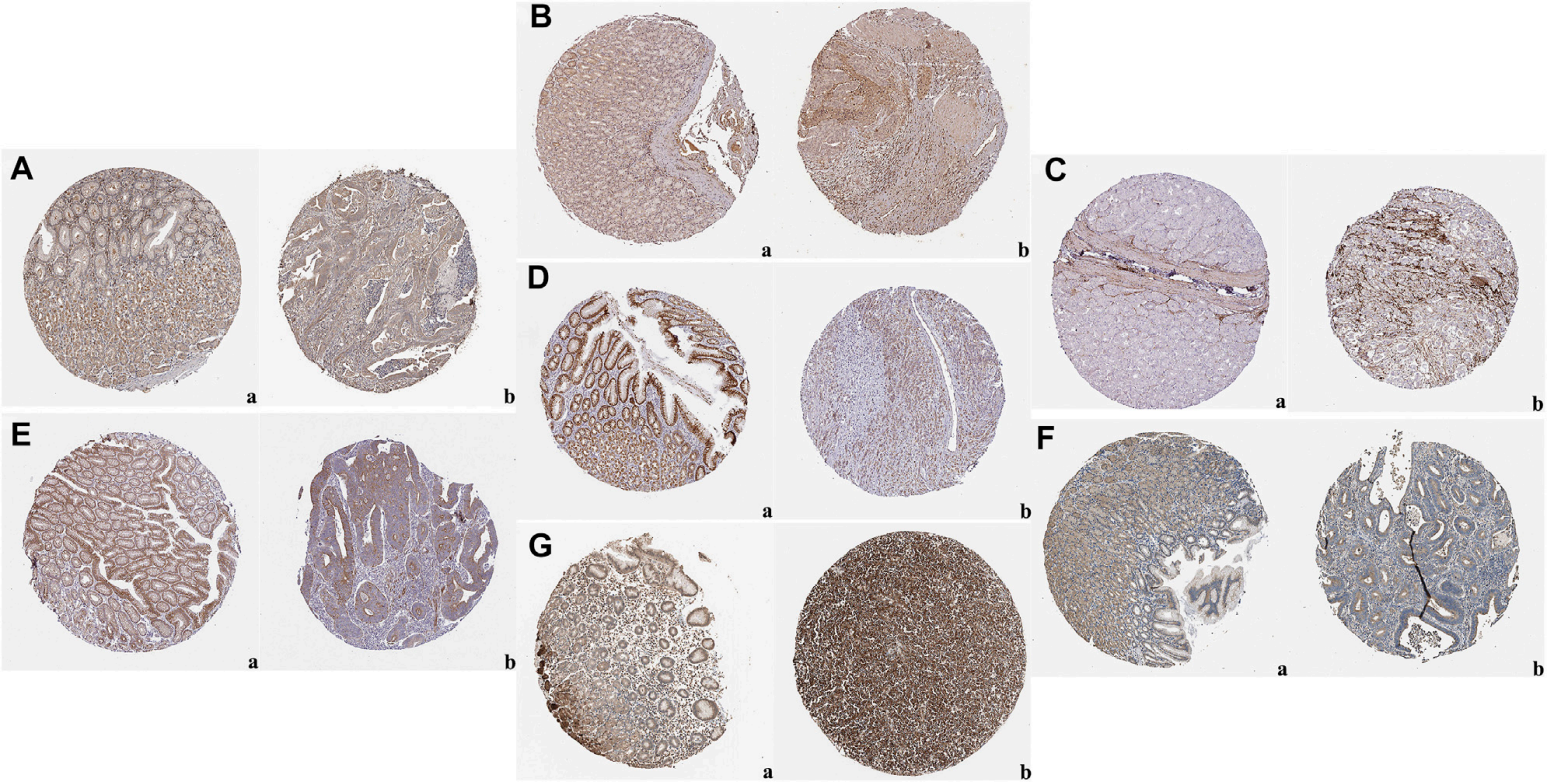
Representative images of immunohistochemical staining of normal gastric tissue (panel a) and gastric cancer tissues (panel b) based on the Human Protein Atlas: **(A)** ADAM Metallopeptidase With Thrombospondin Type 1 Motif 2 (ADAMTS2); **(B)** Collagen Type I Alpha 2 Chain (COL1A2); **(C)** Collagen Type VIII Alpha 1 Chain (COL8A1); **(D)** CTHRC1; **(E)** FAP; **(F)** Thrombospondin 2 (THBS2); **(G)** WNT1-inducible-signaling pathway protein 1 (WISP1).

The expression levels of the up-regulated hub genes between cancer tissues and non-cancer tissues were all statically significant. Expressions of ADAMTS2, COL1A2, COL8A1, COL10A1, COL11A1, CTHRC1, FAP, THBS2, and WISP1 were higher in cancer samples. However, among down-regulated hub genes, there was no statistically significant difference between the expression level of CYP2C9 in cancer samples and normal gastric samples, while the expressions of TFF2 and GIF were statistically significantly lower in cancer samples than normal gastric tissues (Figure 3A). Survival analysis found that higher expressed COL8A1, COL10A1, CTHRC1, and FAP were associated with poor OS. (Figure 3B; Table 1) In addition, the OS of COL8A1, COL10A1, CTHRC1, and FAP verified by Kaplan–Meier plotter firmed their association with worse OS except for FAP. (Figure 4) Immune staining of ADAMTS2, COL1A2, COL8A1, CTHRC1, FAP, THBS2, and WISP1 proteins were shown in Figure 5, however, COL10A1 and COL11A1 were not available from HPA.

**TABLE 1:** Overall survival analysis of Collagen Type VIII Alpha 1 Chain (COL8A1), Collagen Type X Alpha 1 Chain (COL10A1), Collagen Triple Helix Repeat Containing 1 (CTHRC1), and Fibroblast Activation Protein (FAP).

Gene	HR	95% CI	*p* value
COL8A1	0.038	1.42 (1.02–1.97)	0.038
COL10A1	0.033	1.43 (1.03–2.00)	0.033
CTHRC1	0.009	1.56 (1.12–2.17)	0.009
FAP	0.039	1.42 (1.02–1.97)	0.039

### ROC Analysis

**FIGURE 6:**
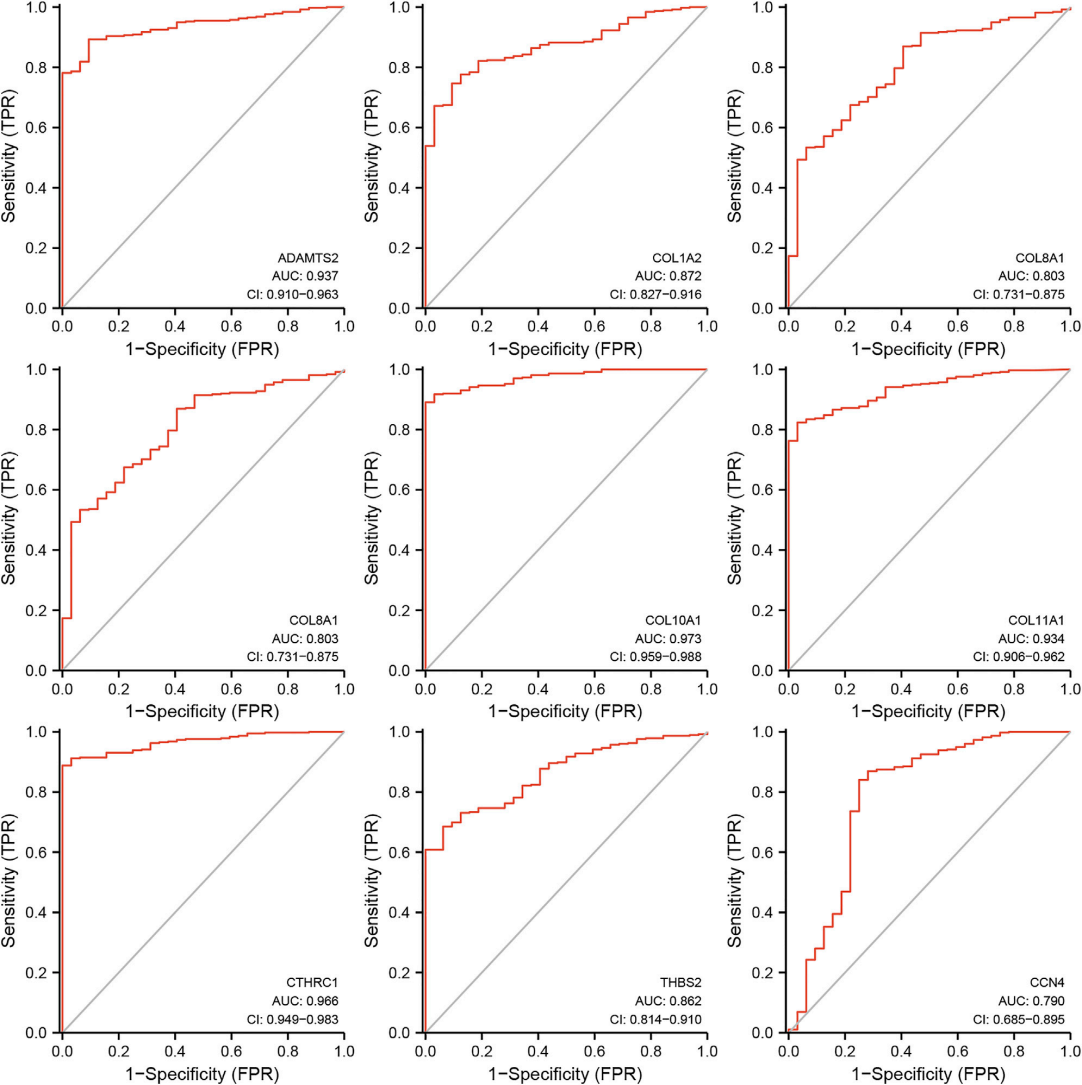
Receiver operating characteristics curve (ROC) curve analysis for the hub genes in gastric cancer.

Further analysis of ROC curve demonstrated that AUC values of ADAMTS2, COL10A1, COL11A1, and CTHRC1 were 0.937 (95% CI: 0.910–0.963), 0.973 (95% CI: 0.959–0.988), 0.934 (95% CI: 0.906–0.962), and 0.966 (95% CI: 0.949–0.983), respectively, (Figure 6). Cutoff values of ADAMTS2, COL10A1, COL11A1, and CTHRC1 were 1.512, 0.382, 0.194, and 2.410.

### Nomogram Development

**FIGURE 7:**
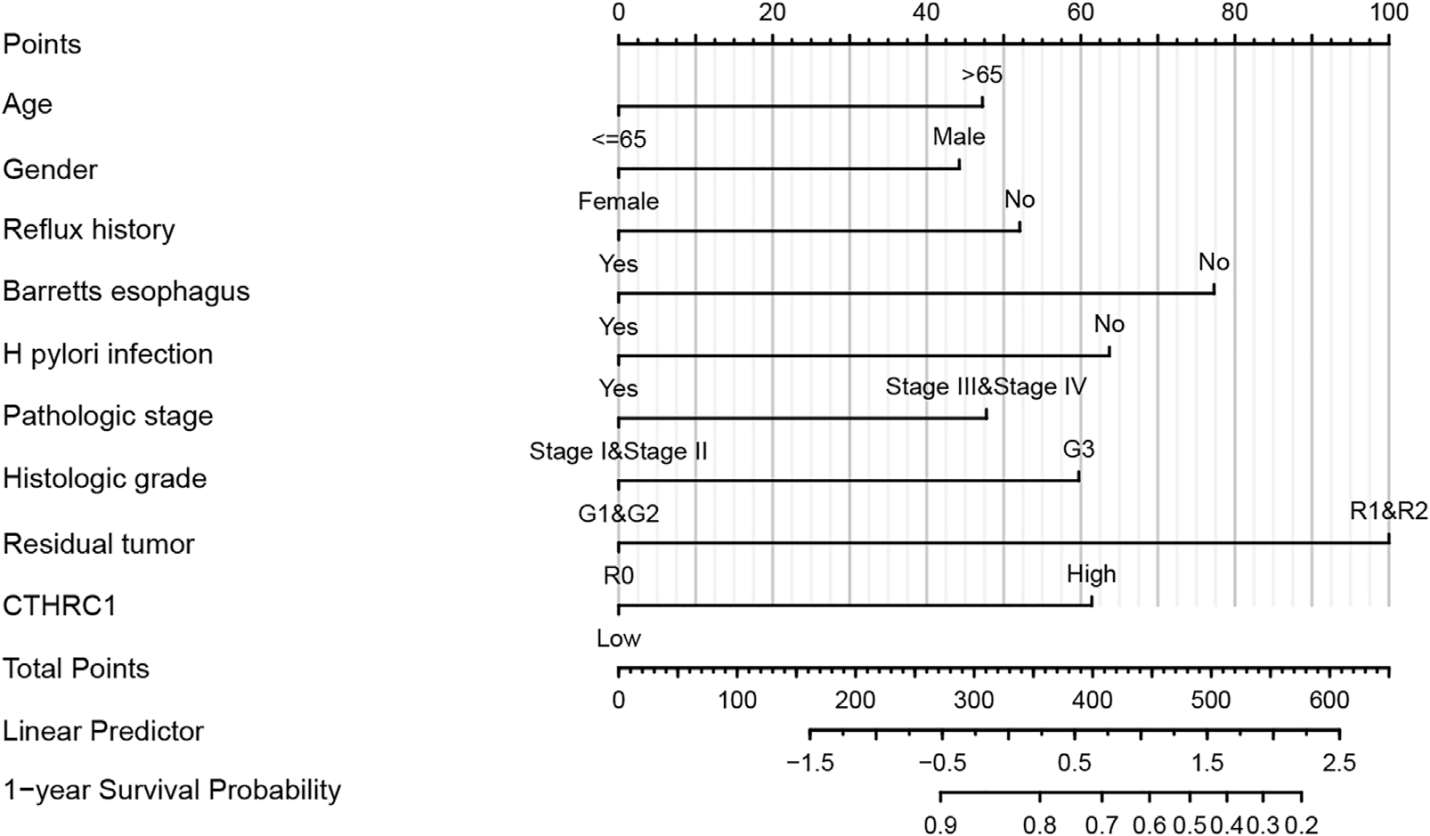
Developed overexpressed CTHRC1 nomogram. Note: The nomogram was developed in the cohort, with age, gender, reflux history, Barret’s esophagus, Helicobacter pylori (*H. Pylori*) infection, pathologic stage, histologic grade, resident tumor. (C-index: 0.709, 95% CI, 0.678–0.740).

A nomogram model incorporating CTHRC1, the hub gene with the worst OS, and other predictors (age, gender, reflux history, Barrett’s esophagus, *H. Pylori* infection, pathologic stage, histologic grade, resident tumor) is shown in Figure 7. The C-index of the nomogram was 0.709 (95% CI, 0.678–0.740).

### Drug Interactions With Hub Genes That Were Associated With Poor Prognosis in LC

As overexpressed COL8A1, COL10A1, CTHRC1, and FAP were found to be associated with worse OS, the top ten drugs or compounds that demonstrated the strongest association with these genes were identified, based on the significance of Spearman correlation. (Table 2)

**TABLE 2:** Top ten drugs associated with COL8A1, COL10A1, and CTHRC1 at expression level.

Gene	Compound	Source	Spearman
COL8A1	1,3-Diphenyl-4-(3-phenyl-4,5-dihydro-1H-pyrazol-5-yl)-1...	CellMiner	0.442684453
	Lovastatin	CellMiner	0.439939641
	tert-Butyl-(2,4-dioxochroman-3-ylidene) methylcarbamate	CellMiner	0.404476742
	Pectenotoxin 1	CellMiner	0.399769302
	Aspiculamycin hcl	CellMiner	0.380594347
	Indole-2,3-dione, 3-[(o-nitrophenyl)hydrazone]	CellMiner	0.308357069
	sri 1,215	CellMiner	0.272899368
	L-685458	CCLE	0.253394313
	Paclitaxel	CCLE	0.249880276
	Sorafenib	CCLE	0.442684453
COL10A1	2-Amino-4-(2-hydroxy-4-methylphenyl)-5-phenylpyrimidine	CellMiner	0.478856028
	Sendanin	CellMiner	0.464032623
	n, o-Diethoxyacetyl-3-demethyldeactylthiocolchicin	CellMiner	0.458826638
	Acetic acid, [1,4,7,10-tetraazacyclododecane-1,7-diyl]bis	CellMiner	0.456711845
	1,8-Naphthyridin-4 (1 h)-one, 2-(3-chlorophenyl)-	CellMiner	0.451246284
	Dihydroartemisinyl ether, stereoisomer of nsc-685988	CellMiner	0.448481534
	1,8-Naphthyridin-4 (1 h)-one, 2-phenyl-	CellMiner	0.446107245
	1-Methyl-3-octadecylimidazolium chloride	CellMiner	0.441177697
	Clanfenur (inn)	CellMiner	0.440695866
	3-Nitro-5-formylisoxazole	CellMiner	0.440318135
CTHRC1	sb-476429-a	CellMiner	0.514263093
	Benzo [1,2-b:4,5-b']dithiophene-4,8-diol, dipropionate	CellMiner	0.442591976
	Benzo [1,2-b:5,4-b']dithiophene-2-carboxaldehyde, 4,8-dioxo-	CellMiner	0.424565795
	1,3,6-Triphenyl-oxazolo (5,4-d)pyrimidin-2',4 (1 h,3 h)-dion	CellMiner	0.423897574
	PF2341066	CCLE	0.268308222
	L-685458	CCLE	0.216083385
	TAK-715	GDSC	0.21088533
	Pelitinib	GDSC	0.190688988
	Daporinad	GDSC	0.179739476
	TAE684	CCLE	0.179349079
FAP	sri 1,215	CellMiner	0.543807
	;N-(benzo[d]thiazol-2-yl)-2-phenyl-7-(3,4,5-trimethoxyph...	CellMiner	0.499632
	b676297k152 3',4'-deoxypsorospermin	CellMiner	0.477193
	Pyrazino [1,2-a]benzimidazole, 1,3-diphenyl-	CellMiner	0.466297
	Okadaic acid	CellMiner	0.46132
	Kinetin riboside	CellMiner	0.457909
	Alsterpaullone	CellMiner	0.428244
	Aspiculamycin hcl	CellMiner	0.420873
	Cyclamin	CellMiner	0.408766
	1,5-Diphenoxyanthraquinone	CellMiner	0.403252

### Go Enrichment and KEGG Pathway Analysis

**FIGURE 8:**
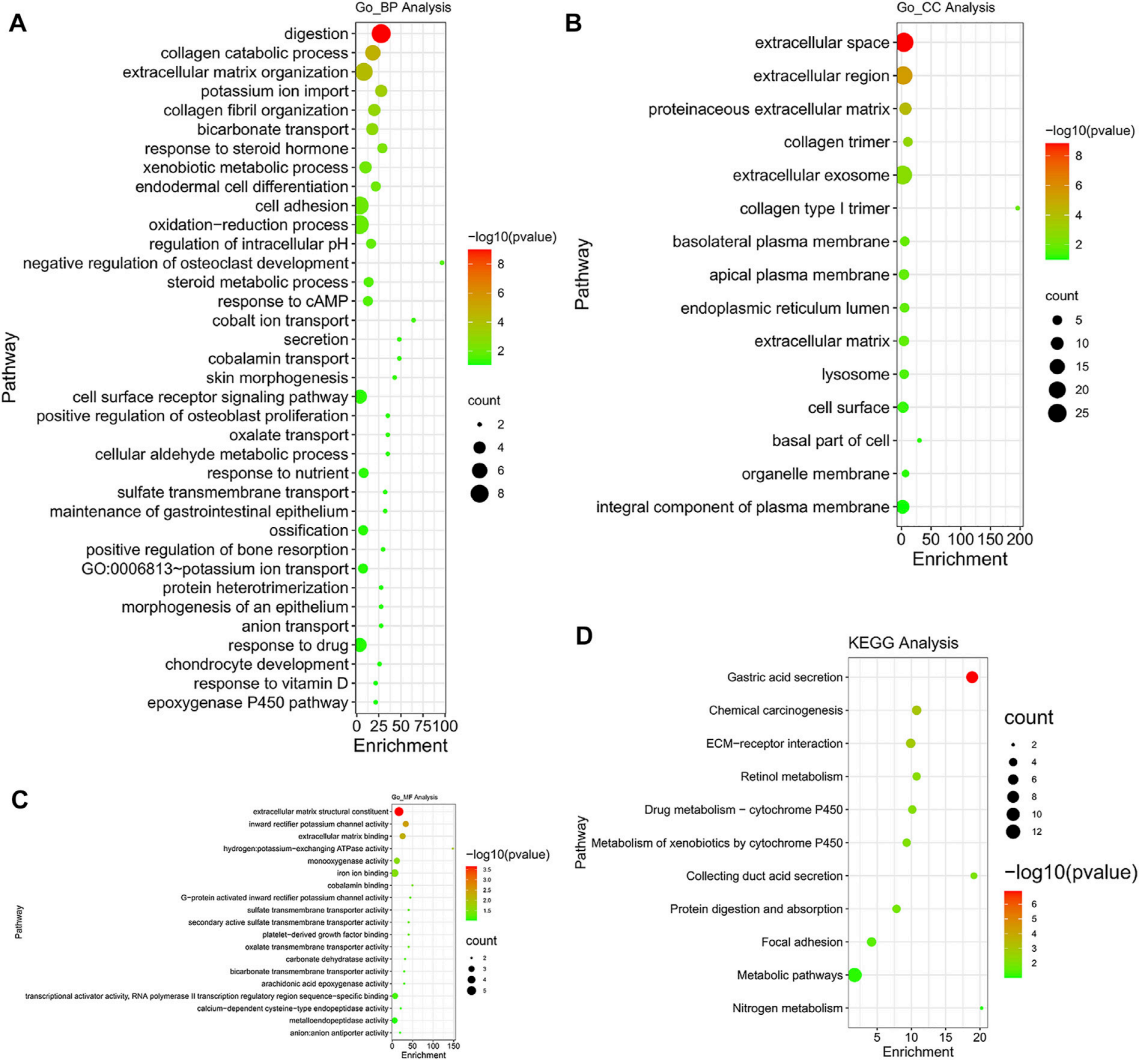
Gene Ontology (GO) enrichment and Kyoto Encyclopedia of Genes and Genomes (KEGG) pathway analysis (DAVID). GO enrichment analysis of target genes based on **(A)** biological process, **(B)** cellular component, and **(C)** molecular function. **(D)** KEGG pathway enrichment analysis of target genes.

Go enrichment and KEGG pathway analysis of the up-regulated and down-regulated overlapping DEGs was conducted by using DAVID and visualized by using bioinformatics online tool (www.bioinformatics.com.cn). Digestion, collagen catabolic process, and extracellular matrix organization were the top three biological processes that were associated with the up-regulated and down-regulated overlapping hub genes (Figure 8A). The extracellular space, extracellular region, and proteinaceous extracellular matrix were the top three major cellular components of these hub genes (Figure 8B). As for molecular function, extracellular matrix structural constituent, inward rectifier potassium channel activity, and extracellular matrix binding were the top three functions (Figure 8C). In regard to KEGG pathways, gastric acid secretion, chemical carcinogenesis, and extracellular matrix (ECM)-receptor interaction were the top three pathways involved (Figure 8D).

### Immune Cell Infiltration of the Hub Genes With Worse Prognosis in GC

**FIGURE 9:**
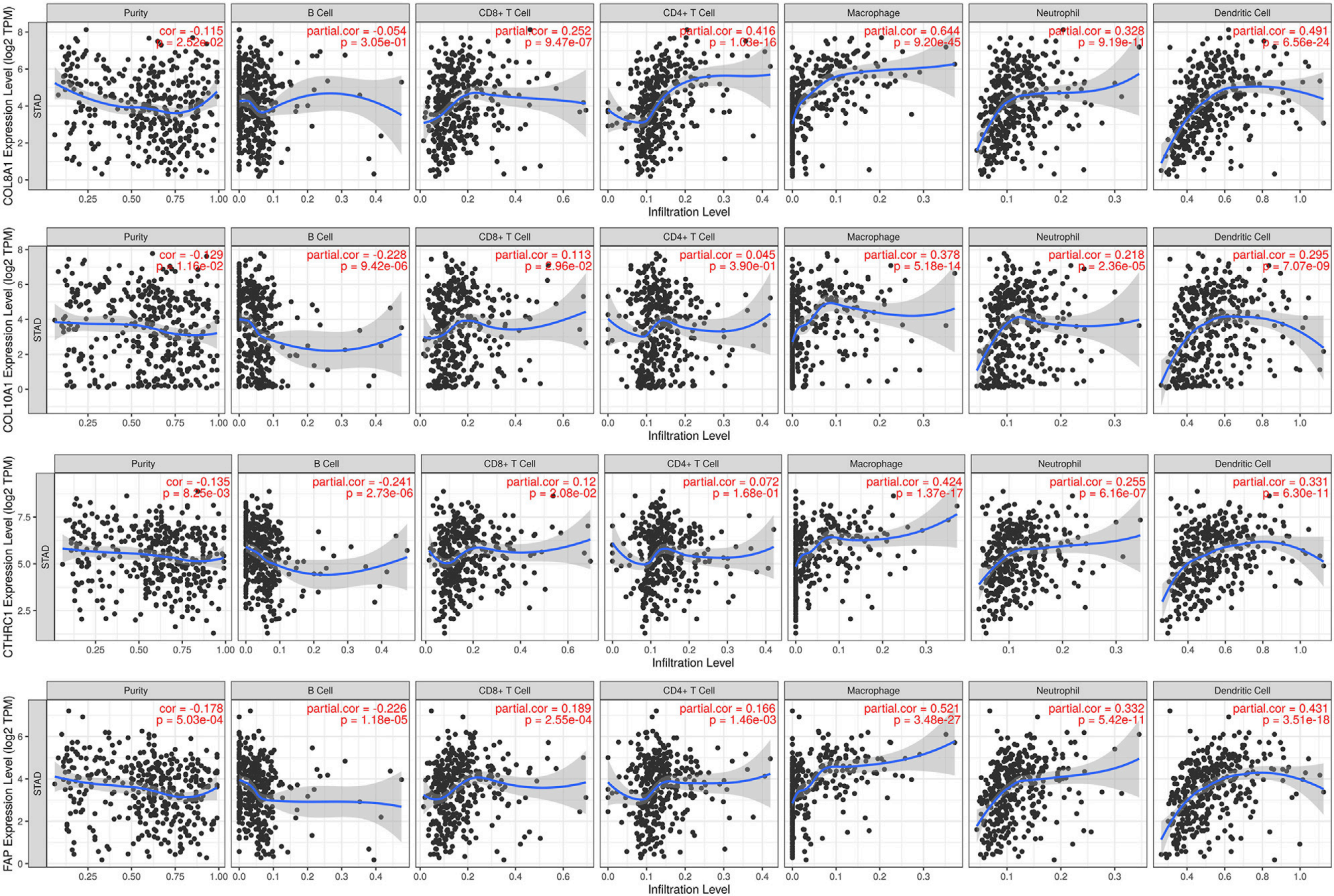
Correlations between COL8A1, COL10A1, CTHRC1, and FAP and immune cell infiltration in gastric cancer (TIMER).

The TIMER database was utilized to investigate the association between COL8A1, COL10A1, CTHRC1, and FAP, and immune cell infiltration, as immune cell levels correlate with the proliferation and progression of cancer cells (Figure 9). The infiltrations of macrophages, neutrophils, and dendritic cells positively correlated with COL8A1, COL10A1, CTHRC1, and FAP. In addition, the Cox proportional hazard model showed that macrophage (*p* = 0.001) and CTHRC1 (*p* = 0.021) were significantly associated with adverse clinical outcomes in GC patients (Table 3).

**TABLE 3:** The Cox proportional hazard model of *COL8A1, COL10A1, CTHRC1, and FAP* and six tumor-infiltrating immune cells in gastric cancer (TIMER).

	Coef	HR	95% CI_l	95% CI_u	*p* value	Sig
B_cell	3.639	38.057	0.661	2,189.983	0.078	
CD8_Tcell	−1.312	0.269	0.016	4.56	0.363	
CD4_Tcell	−2.531	0.08	0.001	8.434	0.287	
Macrophage	5.248	190.1	8.106	4,457.998	0.001	**
Neutrophi	−0.959	0.383	0.002	77.903	0.724	
Dendritic cell	0.736	2.088	0.163	26.73	0.572	
COL8A1	−0.044	0.957	0.799	1.146	0.631	
COL10A1	−0.055	0.946	0.786	1.138	0.557	
CTHRC1	0.294	1.342	1.045	1.723	0.021	*
FAP	−0.06	0.941	0.669	1.325	0.729	
*p < 0.05, **p < 0.01						

### Meta-Analysis

**FIGURE 10:**
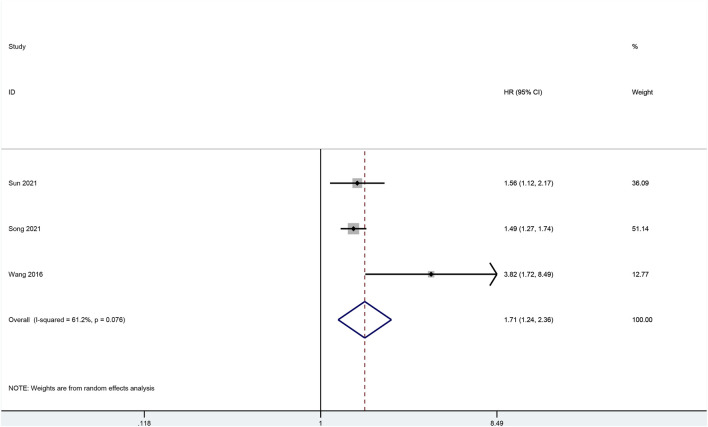
Forrest plot of meta-analysis for CTHRC1.

To verify the results of survival analysis of the hub genes that were associated with worse OS, a meta-analysis was performed. Despite a comprehensive literature search was performed, only three articles investigating CTHRC1 were initially selected for full-text review, while no eligible articles on other hub genes were identified for full-text review. CTHRC1 was associated with the highest HR compared with other hub genes based on our survival analysis, and was shown to have increased HR based on immune cell infiltration analysis; therefore, we pooled the result of the survival analysis of our bioinformatic study with three original articles retrieved from databases (Gu et al., 2014; Wang, 2016a; Dan Song and Zhang, 2021). However, Wang’s article (Wang, 2016b) was excluded due to the incorrect data. The pooled result showed that positive expression of CTHRC1 was associated with poor prognosis of gastric cancer patients. (HR: 1.93, 95% CI: 1.32–2.82, *I* ^*2*^ = 66.1%). (Figure 10) Sensitivity analysis by changing random-effect model to fixed-effect model did not change the result significantly.

## Discussion

As one of the most common malignant tumors and top leading causes of cancer-related death, the GC is induced by a variety of factors (Nagata et al., 2014; Hooi et al., 2017; Research. WCRFAIfC, 2018; Nakanishi et al., 2021; Palrasu et al., 2021; Sung et al., 2021). In this study, 9 up-regulated hub genes (COL1A2, THBS2, COL11A1, COL8A1, COL10A1, ADAMTS2, CTHRC1, FAP, and WISP1) and 3 down-regulated hub genes (TFF2, GIF, and CYP2C9) were identified through integrating 4 gene expression profiles to screen for DEGs and perform PPI network analysis. Previous studies have already identified a number of potential hub genes in GC by using similar research methods. For example, a bioinformatics analysis by Liu et al. (Liu et al., 2018b) found nine hub genes, including DNA Topoisomerase II Alpha (TOP2A), collagen type I alpha 1 chain (COL1A1), COL1A2, NDC80 Kinetochore Complex Component (NDC80), Collagen Type III Alpha 1 Chain (COL3A1), Cyclin Dependent Kinase Inhibitor 3 (CDKN3), Centrosomal Protein 55 (CEP55), Targeting protein for Xklp2 (TPX2), and TIMP Metallopeptidase Inhibitor 1 (TIMP1), that might be associated with the pathogenesis of GC. In addition, the results of the article by Wang et al. (Wang et al., 2020b) identified that Fibronectin 1 (FN1), COL1A1, Inhibin beta-A (INHBA), and cystatin SN (CST1) might be potential biomarkers and therapeutic targets for GC patients. Moreover, Lu et al. (Lu et al., 2021) found 5 key genes, namely, Hyaluronan-Mediated Motility Receptor (HMMR), Cyclin B1(CCNB1), C-X-C motif chemokine ligand 8 (CXCL8), mitotic arrest deficient 2 like 1 (MAD2L1), and Cyclin A2 (CCNA2), in GC patients with poor prognosis using the datasets from GEO database. Additionally, Liu et al. suggested that the expression levels of (ATPase H+/K + Transporting Subunit Alpha (ATP4A), carbonic anhydrase 9 (CA9), Fibrinogen Alpha Chain (FGA), Aldehyde Dehydrogenase 1 Family Member A1 (ALDH1A1), and Ghrelin And Obestatin Prepropeptide (GHRL) were reduced, whereas those of TIMP1, Secreted Phosphoprotein 1 (SPP1), CXCL8, Thy-1 Cell Surface Antigen (THY1), and COL1A1 were increased in GC. Another study demonstrated that COL1A1, Collagen Type V Alpha 2 Chain (COL5A2), Prolyl 4-Hydroxylase Subunit Alpha 3 (P4HA3), and Secreted Protein Acidic And Cysteine Rich (SPARC) showed vital values in prognosis and diagnosis of GC (Niu et al., 2022). Furthermore, another study conducted by Dalkilic to analyze GC transcriptomic data revealed that Secreted Frizzled Related Protein 2 (SFRP2), Early Growth Response 1 (EGR1), Chitinase 3 Like 1 (CHI3L1), COL8A1, Nuclear Enriched Abundant Transcript 1 (NEAT1), INHBA, CXCL8, and Myosin Light Chain 9 (MYL9) were highly expressed, while expression of Gastrin (GAST), GIF, Gastrokine 1 (GKN1), Gastrokine 2 (GKN2), Secretoglobin Family 2A Member 1 (SCGB2A1), and HRAS-like suppressor 2 (HRASLS2) were downregulated (Dalkilic, 2020). Although a variation of hub genes were found in previous studies, our study added additional evidence of potential hub genes in GC that could be served as important biomarkers.

As expected, GO analysis found that all the hub genes were related to the digestion pathway for the biological process. Collagen catabolic process and extracellular matrix were also involved significantly in the biological process. For cellular components, the extracellular space, and extracellular regions, as well as proteinaceous extracellular matrix, were mostly involved. In regards to molecular functions, extracellular matrix structural constituent, inward rectifier potassium channel activity, and extracellular matrix binding were greatly involved. KEGG analysis identified that the gastric acid secretion pathway was significantly involved, which was unsurprising given that interference with gastric acid secretion may have caused damage to gastric mucosa. In addition, chemical carcinogenesis and ECM-receptor interaction were also found to be involved. Most of the pathways found in GO and KEGG analyses were closely related to either gastric functions or extracellular activities and structures. A summary of these biological functions and pathways that the hub genes were mostly involved in are shown in Table 4.

**TABLE 4:** Biological functions and pathways that the hub genes are mostly involved in.

Category	Pathway
Biological processes	Digestion
	Collagen catabolic process
	Extracellular matrix organization
Cellular components	Extracellular space
	Extracellular region
	Proteinaceous extracellular matrix
Molecular functions	Extracellular matrix structural constituent
	Inward rectifier potassium channel activity
	Extracellular matrix binding
KEGG pathway	Gastric acid secretion
	Chemical carcinogenesis
	ECM-receptor interaction

Further validation of these hub genes found that all up-regulated hub genes were expressed higher in GC tissues, while among the down-regulated hub genes, the expression level of CYP2C9 was not significantly lower in GC samples. Further survival analysis of the up-regulated hub genes found that COL8A1, COL10A1, CTHRC1, and FAP were associated with poor OS. COL8A1 was known to encode the alpha 1 chain of collagen, type VIII, and thus may modulate migration, proliferation, and adherence of various cells (Zhao et al., 2009a). COL8A1 was also proposed to promote the migration of certain cancer cells by mediating ECM-receptor interaction (Peng et al., 2020). Zhao et al. showed that knockdown of COL8A1 induced the inhibition of hepatocellular carcinoma growth and invasion (Zhao et al., 2009a). Previous studies also found that COL8A1 was associated with poor prognosis of GC, consistent with our findings (Wu et al., 2020a; Chen et al., 2020; Wang et al., 2020c; Zhou et al., 2020). COL10A1, which encodes Collagen type X alpha 1, was found to be overexpressed in different types of cancer, such as esophageal cancer and breast cancer, and promoted the malignant progression by upregulating the expression of Prolyl 4-hydroxylase beta polypeptide (P4HB) (Song et al., 2021; Yang et al., 2021). As a member of the collagen family, COL10A1could activate ECM remodeling and the epithelial-to-mesenchymal transition (EMT), vascular endothelial growth factor receptor 3 (VEGFR3) and Wnt signaling pathway, and its aberrant expression might affect the development of cancer (Song et al., 2021). A recent study identified COL10A1 as a potential inducer of EMT (Li et al., 2018b). Silencing of COL10A1 was found to induce inhibition of cell proliferation, migration, and invasion in GC (Li et al., 2020). Consistent with previous studies, we also found that COL10A1 was associated with adverse outcomes in GC (Chen et al., 2021). Collagen triple helix repeat containing-1 (CTHRC1) was known as a cancer-related protein, and overexpression of CTHRC1 was believed to be involved in tumorigenesis, proliferation, invasion, and metastasis in various gastrointestinal malignancies, including gastric cancer, as well as other non-gastrointestinal malignant tumors (Wang et al., 2012b; Mei et al., 2020). A recent *in vitro* study found that CTHRC1 increased CXCR4 expression through upregulating hypoxia-inducible factor 1-alpha (HIF-1α) expression, leading to the promotion of cell migration and invasion in GC (Ding et al., 2020). Another study found that repression of CTHRC1 protein activity could inhibit cell proliferation, migration, and invasion in GC (Yu et al., 2015). In addition, a previous clinical study also concluded that higher expression of CTHRC1 was associated with worse prognosis (Gu et al., 2014). Moreover, the results of our meta-analysis further confirmed that CTHRC1 overexpression was associated with worse OS. As for FAP, it is one of the active members of the S9b protease family and known to promote EMT of oral squamous cell carcinoma (OSCC) (Wu et al., 2020b; Huang et al., 2021). However, comprehensive research of FAP on GC is limited. Our finding of higher expression of FAP in GC and its association with worse OS suggests that more studies are needed to investigate the mechanism and effects of FAP in GC. It also has to be noted that the verification by Kaplan–Meier plotter only found marginal association with worse OS, and thus more original studies on its prognostic role in GC are also needed.

As CTHRC1 was found to have the highest HR among other up-regulated hub genes, a nomogram was created based on CTHRC1, age, gender, reflux history, Barrett’s esophagus, *H. Pylori* infection, pathologic stage, histologic grade, and resident tumor. This showed a relatively high accuracy of prediction given its C-index above 0.7. Thus, CTHRCI demonstrated its utility as not only a diagnostic biomarker, but also as one of the predictors in the nomogram developed in this study.

Further analysis of the up-regulated hub genes also identified the potential diagnostic markers found that ADAMTS2, COL10A1, COL11A1, and CTHRC1 as potential diagnostic markers given that their AUC value and 95% CI were all above 0.9. There were a few studies on the prognostic value of ADAMTS2 in GC, but the evidence of its diagnostic value on GC is limited (Jiang et al., 2019; Liang et al., 2020), while previous study showed that high levels of COL10A1 in plasma could provide diagnostic value in GC with AUC of 0.9171 (*p* = 0.0002) (Necula et al., 2020). As for COL11A1, a previous study suggested it could be used for differentiating the malignant lesions from premalignant tissues in stomach cancer based on 42 tissues samples (Zhao et al., 2009b), and our result further supports their conclusions though more studies are still warranted. Regarding CTHRC1, although its prognostic predicting value was more extensively studied, its diagnostic value has not been fully investigated and our results provide new evidence of its potential use in diagnosis of GC.

To further explore the potential drugs that may target the hub genes that are associated with worse prognosis, analysis of drug interactions was performed. For COL8A1, the compound or drug that showed the highest interaction was not investigated previously on GC. The second strongest interactor was lovastatin, an agonist of Src homology-2 domain-containing protein tyrosine phosphatase-2 (SHP2) that was found to significantly enhance the efficacy of chemotherapy in colon cancer (Wei et al., 2021). Other *in vitro* studies found that lovastatin inhibited gastric cancer cells (Cheng-Qian et al., 2014; Zhang et al., 2019b). As for COL10A1, although the top interacting compound, 2-amino-4-(2-hydroxy-4-methylphenyl)-5-phenylpyrimidine, was not previously investigated, the second drug on the list was sendanin. This compound was shown to inhibit the cancer cell lines according to previous *in vitro* study (Kim et al., 1994). In regards to CTHRC1, the first four compounds on the list were not well studied on cancer, while PF2341066, an inhibitor of anaplastic lymphoma kinase and c-Met later named as crizotinib, was previously shown to have antitumor activity of PF-2341066 in experimental models of anaplastic large-cell lymphoma. Its clinical use is increasingly reported in gastric cancer patients (Christensen et al., 2007; Sabree et al., 2018; Hou et al., 2019; Aparicio et al., 2021). Regarding FAP, although the first few drugs on the list were not well studied, kinetin riboside was found to inhibit colon cancer cells or even stimulated apoptosis (Cheong et al., 2009; Dudzik et al., 2011; Rajabi et al., 2012). Despite a number of studies investigating the compounds or drugs found in our analysis, more research studying on their effects on GC patients is still needed.

Further analysis on the relationship between COL8A1, COL10A1, CTHRC1, and FAP, and immune cell infiltrations in GC found that macrophages, neutrophils, and dendritic cells are positively correlated with these genes. Tumor-infiltrating macrophages were known to play a vital role in tumorigenesis by promoting tumor growth, migration, and invasion, as well as suppression of anti-tumor activity and progression (Van Overmeire et al., 2014; Rihawi et al., 2021). Moreover, tumor-associated macrophages (TAMs) were proposed to have the ability of significantly interfering with treatment response to chemotherapy, immune checkpoint inhibitors (ICIs), antiangiogenic drugs, and even radiotherapy or other treatment methods, leading to failure of treatment (Mantovani and Allavena, 2015; Qiu et al., 2018; Rihawi et al., 2021). Previous studies based on gastric cancer samples found increased neutrophil infiltration in GC, which is consistent with our finding (Kim et al., 2017). It was also shown that neutrophils activated by granulocyte-macrophage colony-stimulating factor (GM-CSF) could express CD54 and B7-H4 that are associated with reduced overall survival of GC patients following surgery (Shan et al., 2021). Tumor-associated neutrophils (TANs) were proposed to induce lymphangiogenesis and angiogenesis, and it was shown that local infiltration of certain types of TANs may play a role in the metastasis in GC (Hiramatsu et al., 2018). Infiltration of dendritic cells in gastric cancer and lymph nodes of GC patients are well known (Tsujitani et al., 1992; Tsujitani et al., 1995), and its infiltrating level in GC was also found to be closely correlated with macrophage infiltration (Xiang et al., 2020). One study found that higher level of dendritic cell infiltration was associated with longer OS (Higgins and Thompson, 2002; Hu et al., 2014). Given that our findings of the roles of the tumor-infiltrating immune cells and the selected up-regulated hub genes in the tumor microenvironment, further investigation and more comprehensive studies on the associations of tumor-infiltrating immune cells and these genes in GC are needed.

This bioinformatic study also has some limitations: First, all data were retrieved from online databases; therefore, the results need to be validated with other cohorts and experiments. Second, as this study mainly aimed to explore the potential clinical values of selected hub genes in the diagnosis and therapy of GC, the details of their mechanisms were not comprehensively explored, especially FAP with very limited number of previous studies of its effect on GC. Third, the TIMER database was mainly based on the TCGA database; therefore, the results need to be verified in the future with other cohorts and experiments.

## Conclusion

In conclusion, among the nine up-regulated hub genes (COL1A2, THBS2, COL11A1, COL8A1, COL10A1, ADAMTS2, CTHRC1, FAP, and WISP1) and three down-regulated hub genes (TFF2, GIF, and CYP2C9), ADAMTS2, COL10A1, COL11A1, and CTHRC1 have demonstrated potential as diagnostic markers. COL8A1, COL10A1, CTHRC1, and FAP are associated with worse prognosis and could be potential prognostic biomarkers and therapeutic targets. The infiltration of macrophages, neutrophils, and dendritic cells are positively correlated with these COL8A1, COL10A1, CTHRC1, and FAP, suggesting the need for exploration of their roles in the tumor microenvironment of GC. Among these hub genes, CTHRC1 was found to have the highest prognostic accuracy and associated with worst prognosis as compared with other hub genes, and could be the next research hot spot. A nomogram based on CTHRC1 and other clinical predictors might be useful in clinical decision-making.

## Data Availability

The datasets presented in this study can be found in online repositories. The names of the repository/repositories and accession number(s) can be found in the article/Supplementary Material.
